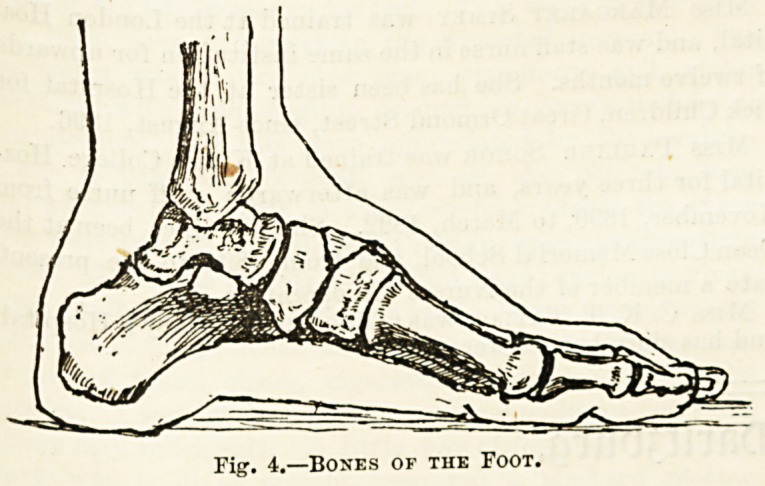# "The Hospital" Nursing Mirror

**Published:** 1900-03-31

**Authors:** 


### "The Hospital" Nursing Mirror.

Being the Nursing Section of "The Hospital."

[Contributions for this Section of "The Hospital" should be addressed to the Editor, The Hospital, 28 & 29, Southampton Street, Strand, London, W.C., and should have the word "Nursing" plainly written in left-hand top corner of the envelope.]

### Notes on News from the Nursing World.

### THE PRINCESS OF WALES AND HER HOSPITAL SHIP.

In an article headed "Netley Patients," which appeared in *Lloyd's Newspaper* on Sunday, publicity was given to a statement alleged to have been made by Sergeant Harper, of the Argyll and Sutherland Highlanders, who, according to our contemporary, "put the case pretty strongly, evidently feeling the truth of every word he uttered."

"We had," he is reported to have said, "everything we wanted when we were 'down' in South Africa; but since we left Table Bay I haven't known what a square meal is. I came home in the 'Princess of Wales'—there she lies, and he pointed to the vessel at anchor in the placid Southampton Water, 'and it's a fact, it's the worst ship for food I ever set foot in. From half-past four in the afternoon till eight o'clock the next morning, every day while we were coming over, we never had anything to eat, and generally we were without a smoke. We never had sugar in our tea; to get salt we had to wait half an hour; and, to speak the truth, I heard more complaints on that one voyage in the 'Princess of Wales' than I heard during seven years I spent in the army."

From our own knowledge we were satisfied that the serious complaint imputed to Sergeant Harper was utterly without foundation; but, in order to settle the question beyond doubt, we enclosed the article to the Princess of Wales, drawing the attention of her Royal Highness to the allegations. We have received the following reply: "Marlborough House, Pall Mall, S.W., 26th March, 1900. Sir,—I am directed to reply to the letter you have addressed to the Princess of Wales, forwarding for her Royal Highness's perusal a cutting from *Lloyd's Newspaper*. I have just had an interview with Major Morgan, R.A.M.C., the medical officer in charge of the hospital ship 'Princess of Wales,' which has had to put back to England in consequence of damage to boiler by an explosion. He denies *in toto* the statements of Sergeant Harper, and says he never had any complaint of insufficient amount of food. Of course, some of the invalids—those who had been suffering from dysentery or enteric—were on hospital diet, and were not allowed the same amount as those who had not had these diseases. Possibly Sergeant Harper may have been one of these, but without reference to the case book Major Morgan could not say. He himself used to serve out cigars and cigarettes to all who wanted them. I may add I am quite sure there could have been no lack of comforts and extras supplied to all who came home in the 'Princess of Wales.' I remain, sir, yours faithfully, S. de A. C. Clarke, Major-General." Comment on this letter is superfluous, though we may add that the testimony of the nurses on board the "Princess of Wales" Hospital Ship, that there were no complaints from the patients, is entirely in accord with that of Major Morgan.

### THE PRINCE OF WALES AND THE WELSH NURSES.

The Prince of Wales will inspect the staff and nurses of the Welsh Hospital for South Africa at Marlborough House on Monday next at twelve o'clock. The matron of the hospital is Miss Marion Lloyd, who is well known in the nursing world. Miss Lloyd was trained at the General Hospital, Bristol, for three years, where she was afterwards staff nurse for two years. From October, 1893, to September, 1894, she was first night superintendent and then deputy matron at the Royal Albert Edward Infirmary, Wigan. In September, 1894, she became matron of the Cancer Hospital, Manchester, and since May, 1898, she has been matron at Bolton Hospital. At a dinner given the other evening to Professor Thomas Jones, who is in charge of the Welsh Hospital, it was intimated that all the nurses are able to converse in Welsh.

### A HIGHLY FAVOURED INSTITUTION.

The nursing staff for the Edinburgh and East of Scotland Hospital in South Africa have appropriately been chosen from Edinburgh. The whole of the seven nurses—Miss A. W. Gill (superintendent), Miss J. Cameron, Miss E. M. Herriet, Miss A. B. Cameron, Miss E. Cumming, Miss L. Boyd, and Miss J. Galloway were trained at the Royal Infirmary, Edinburgh. Miss A. B. Cameron, who was nurse for a time at the Longmore Hospital for Incurables, and Miss Cumming, who has been matron of the Nurses' Home of Rest, are the only two who have had any outside experience. The nurses, in company with Professor Chiene and the medical staff of the hospital, attended St. Giles's Cathedral, Edinburgh, on the Sunday before their departure, and the preacher, Dr. Marshal, made special reference to those who are about to leave on such a noble mission, and especially to the nurses.

### THE RATIONS OF NAVAL NURSES.

At present naval nurses are always men. This is a matter for regret, but it is no reason why the rations should be inadequate. Men require sufficient food as much as women, and the complaint of a naval nurse, who owns to the possession of a good appetite, that he is not allowed any refreshments when he is on night duty nursing the sick, unless he chooses to pay for them, is reasonable. We recognise, also, the force of the plea that the hungry nurse may be tempted to share the rations of the sick patient, without asking the consent of the latter. The Admiralty can really afford to remove this temptation by making the scale of rations to the nurses more liberal.

### DRESS AT A NURSES' DANCE.

Of the new nurses' home which has been added to the Wandsworth and Clapham Infirmary there is nothing but good to be said. It is three storeys in height, of very handsome character, with all the most recent improvements in sanitation, and contains accommodation for 74 nurses. The cost has been about £15,000; and, as Canon Erskine Clarke, at the opening ceremony, said: "The money is well spent if it places a staff of zealous nurses in conditions of health and comfort which will enable them to perform their work with more pleasant surroundings." But if anything were wanted to prove that dancing at infirmaries is a mistake, it would be a report given in a local paper of the proceedings at the fifth annual ball of the nursing staff of the infirmary. We give the following extract:—

"A few gentlemen, experts in feminine charms and graces, undertook the task of selecting the belle of the evening, rather a difficult one in the midst of such an array of youth and beauty, assisted by the art of the dressmaker and other artistes known only to ladies. The choice, however, was given to Probationer Crosby, who wore a cream-coloured dress, relieved at the waist with a heliotrope sash. Probationer Parry was given as a good second, in a white satin dress, which hung in graceful folds, the body being trimmed with a trellis-work of green velvet. Probationer Button also deserves mention, her pretty pink dress being greatly admired. Nurse Lears looked charming in light green silk, as did also Nurse Clears, whose stately figure showed to advantage in white satin and silver trimmings. Sister Gregory, who wore a charming pale blue silk dress, which blended well with her brunette beauty, was much admired."

One wonders on reading this account of the dresses, which might have been taken from the columns of a so-called society paper, how often for weeks before the event the thoughts of the nurses were with their patients, and how often they were mentally engaged in elaborating their costumes.

### "NURSE-PHOBIA" IN THE BOARD-ROOM OF THE BRISTOL GUARDIANS.

The other day the Bristol Board of Guardians abolished the title of sister in their infirmary, and some of them have now been trying to prevent the nurses in their employ from having three weeks' holiday. Canon Griffiths, in commenting upon the attempt and the speech of Mr. Ring, who said, *apropos* of nothing, that "thousands of tradesmen's wives had to be content with two or three days' holiday a year," averred that "nurse-phobia" was rampant at the board. We are glad to see, however, that the complaint does not extend to lady guardians, one of whom, bearing the honoured name of Nightingale, appealed, in the interests of the patients, that the three weeks which was implied in the rescinding should be given. It was good, she contended, that the nurses should have such relaxation as would enable them to return to their duties with their health re-established. Happily, her appeal was successful, and it may be hoped that the fit of "nurse-phobia" is already passing away.

### NURSING IN NAZARETH.

Considering how many British hospitals there are now in Palestine it seems curious that the small Galilean town where for 30 years the great Physician lived should not possess one. Yet such is the case in Nazareth. Some little time ago the Edinburgh Medical Mission tried to establish a hospital there, and the building was commenced. But the Turkish Government threw so many obstacles in the way and demanded so much "backsheesh" that ultimately, though with much reluctance, the scheme had to be abandoned, and the house intended for the hospital is now used as a barracks. The Scotch doctor belonging to the mission has a dispensary in the town, where he attends out-patients; but there is no British accommodation for in-patients, which is very sad. Still, the natives are not without skilled nursing in Christian hospitals, and though they may not be British institutions, they are at any rate European. At the temporary Austrian hospital there is at present room for about eight patients, but a large permanent hospital is being built, and will have accommodation for at least 50 people. From all accounts and appearances it will be quite modern, with an up-to-date operating theatre. This hospital only receives men and boy patients. It belongs to a Latin brotherhood, comprising a priest, a doctor, a dispenser, and two male nnurses. It has no women workers of any kind. In the town the Sisters of Charity (Latins) have a good hospital, with accommodation for some 18 or 20 patients. It is kept in beautiful order, and the beds are all arranged with the utmost care and with regard to the comfort of the sick person. Over each bed is a shelf where the drinking water-bottle and other things stand. It is in some respects preferable to the usual method of keeping such things—particularly in the East—on the locker, mantel-piece, or the window-sill. The wards are bright and sunny, and the sisters—who have been trained in French hospitals—are capable women. This hospital has a dispensary, where twice a week many out-patients receive medicine. The doctor is a clever, qualified Syrian. Each hospital is cosmopolitan. Patients are taken gratuitously, and irrespective of religion.

### PLAISTOW MATERNITY CHARITY.

The annual general meeting of the Plaistow Maternity Charity and District Nurses' Home was held on Tuesday afternoon, by the kindness of the Duchess of Sutherland, at Stafford House, but owing to indisposition her Grace was unable to preside as she had promised. The Bishop of Colchester took the chair in her stead. The report showed that 2,061 maternity patients had been attended in their own homes, necessitating 36,400 visits from the nurses; that 2,530 patients suffering from general complaints had received 83,066 ordinary visits and 4,658 special visits; whilst there were 2,422 out-patients who made 14,327 attendances at the nurses' home. The number of women trained as district, village, maternity, or cottage nurses was 123. The total ordinary income for the year amounted to £4,335, the ordinary expenditure to £4,413, a deficit of £78. There is a deficit of £536 for the last three years. The first resolution, pledging the meeting to take care that the claims of this charity should not be overlooked in consequence of newer calls upon the public purse, was moved by Mr. English Harrison, and seconded by the Rev. T. H. Gilbert (delegate of the West Ham Men's Guild). The second, thanking the honorary consulting and other hon. medical officers for their constant and ready help, was proposed by Mr. A. Pritchard, seconded by the Rev. P. M. Bane, and supported by Mr. Gardiner; and the final resolution, thanking the Duke and Duchess of Sutherland for the use of Stafford House for the occasion, was moved by the Archdeacon of Essex, and seconded by Mr. Hurst.

### NO PIANO FOR THE WAKEFIELD INFIRMARY NURSES.

The Wakefield Guardians recently had under discussion the advisability of providing a piano for the use of their infirmary nurses. The chairman recommended the purchase of an instrument which could be obtained for the sum of £28 10s. One of the guardians considered the idea "most extravagant," and said that the Board ought to save £28 rather than spend it on a piano. It might, he contended, prove a nuisance, and attract attention which ought to be devoted elsewhere, and he moved that the item referring to a piano should be left out of the minutes. Other members concurred in his sentiments, and the amendment was carried by 14 votes to 13. It is somewhat surprising that although scarcity of nurses is the cry of so many boards of guardians, they often begrudge any trifling expenditure entailed in making the lives of their nurses more bright and cheerful.

### THE NEW DEPARTURE AT THE BRITISH LYING-IN HOSPITAL.

Many improvements have been made during the year at the British Lying-In Hospital, Endell Street, both in the hall and dining-room and in the wards, which look complete with curtains and screens of a soft green colour. Both mothers and babies seem snug and happy, and the Duchess of Portland was very pleased with their appearance and the arrangements made for their welfare on the occasion of her visit. The matron is much gratified that many friends interested in the hospital take advantage of her Wednesday afternoon's "At Home." She will welcome anyone who takes an interest in maternity work. There are a great number of candidates seeking training as nurses, and she is delighted that in future all untrained women must undergo a two months' course of teaching, instead of one month as hitherto. This innovation was decided upon last December, and is now coming into force. Numerous pupil midwives have been prepared for the L.O.S. examination, and all presenting themselves obtained the certificate.

### WARRINGTON GUARDIANS AND THE WARRINGTON NURSING ASSOCIATION.

A fortnight ago we expressed our regret that the Warrington Guardians had refused to make a small increase in their subscription to the Warrington District Nursing Association. The subject is attracting a great deal of attention in the town of Warrington, and the statement made by one of the Guardians as a plea for the penurious policy of the Board that "the benefits of the nursing association are confined to the middle classes," has excited considerable indignation. Miss F. E. Whitfield, the superintendent of the association, states that the cases on the books at present "represent anything but the middle classes, and reveal a succession of heart-rending histories of suffering and poverty." We are not at all disposed to find fault with the Guardians for spending money in the erection of a new hospital at a cost of nearly £40,000; but, by the side of this expenditure, their objection to subscribe another paltry £5 a year to an association which has admittedly been the means of saving many lives and preventing much pauperism in Warrington, is extraordinary and indefensible.

### A HOME FOR NURSES AT NEWPORT, SALOP.

There has just been opened at Newport, Salop, a home for the nurses employed in the town and neighbouring parishes. Hitherto the nurses had lodged in the parishes where they chiefly laboured, but it was thought desirable that there should be a central home where they could reside when not on active duty. The home which has been bought, repaired, and furnished by the generosity of Miss Roddam, contains ample accommodation for the matron and nursing staff, and great satisfaction has been expressed with all the arrangements.

### NURSING AT ARARAT HOSPITAL.

At a recent meeting of the Committee of the Ararat Hospital, the Resident Surgeon stated that by adopting a system found to work most satisfactorily in other places of having a dispensing nurse acting under his supervision, in the place of a head wardsman, he could increase the rate of salaries, prevent the drifting away of qualified nurses, and secure a higher efficiency. In fact, the present policy of the hospital is, by making the hours the shortest and the salaries the highest, in the colony, to attract the best nurses; and it seems to be succeeding as admirably in that distant part of the world as it does at home.

### WHAT CONSTITUTES A TRAINED NURSE?

A nurse, having recently taken a fresh case, after being on duty all night and until two o'clock the next day, began to wonder if any arrangements had been made for her to go to bed. Turning to a relative of the patient's she made some remark about it, when, to her astonishment, the lady to whom she had spoken rejoined in shocked tones, "But, nurse, I thought you were *trained!*" Evidently the idea of this good lady is that the training of a nurse proceeds on similar lines to that of an athlete, and includes a training in doing without sleep.

### SHORT ITEMS.

The third annual report of the Northumberland County Nursing Association contains the interesting information that the Duke of Northumberland has offered to contribute £10 a year for two years towards the maintenance of a nurse in any new affiliated districts wishing to start a nurse in the year 1900, providing they be recommended to him by the Managing Committee of the County Nursing Association, and providing that the remainder of the sum necessary for their annual maintenance be raised locally within the year.—A very satisfactory report was submitted at the annual meeting of the Worthing District Nursing Association, the number of visits paid by the nurses in 1899 being 6,021, and the balance of receipts over expenditure £65.—The Palestine and Lebanon Nurses' Mission, of which the Rev. F. Paynter is president, are appealing to the public for a sum of £200 to build a men's ward in the hospital at Baakleen, where a staff of trained nurses are engaged in work amongst the Druses of Lebanon.—Miss C. A. Little, M.R.B.N.A., is about to form a nurses' co-operation at 59, Spring Bank, Hull, in connection with a nursing home. The object is to help the nurse to obtain the full amount of her earnings, minus a percentage, and to secure for the public the services of the fully-trained nurse.—The first annual dinner of the Central Hackney Musical and Benevolent Society—which has for its object the assistance of the North-Eastern Hospital for Children—was very successfully held last week at the Holborn Restaurant, under the able presidency of Mr. M. Siegenberg.—The Hon. Secretary of the Children's Penny Fund states that it was the Princess Christian, and not the Princess Victoria of Schleswig-Holstein, who started that fund.

### Lectures on Nursing for Probationers.

By E. MacDowel Cosgrave, M.D., &c., Lecturer to the Dublin Metropolitan Technical School for Nurses.

### No. II.—THE LIMBS.

The limbs are built up on the same plan, each being divided into three parts, named respectively upper-arm, fore-arm and hand, and thigh, leg, and foot.

The upper limbs are attached to the shoulder-girdle, composed on each side of a shoulder-blade, or scapula, which is the flat bone slung loosely by muscles at the back of the thorax, a widened-out part being hollowed to form the socket of the shoulder-joint; and of a collar-bone, or clavicle, stretching from the sternum or breast-bone to the scapula, keeping the arm held out from the thorax, and preventing the shoulders being drawn forwards, narrowing the chest.

Fig. 2.
Bones of Arm.

Fig. 3.
Bones of the Lower Extremity.

The shoulder-girdle is loosely attached, and by its movements increases the range of movement of the arm.

The lower limbs are attached to the hip-girdle, composed at each side of the pubes in front, the illium at the side and the ischium below (the three together being called the os innominatum); these are fused with the sacrum or lower end of the spine into a solid basin—the pelvis. The socket of the hip-joint is deep and immovable.

The upper-arm contains a single bone, the humerus; its rounded head fits into the hollow of the scapula, forming with it a ball and socket joint. In the fore-arm are the ulna and radius, the former is at the outside running towards the little finger; the latter at the inside running towards the thumb. The ulna joins the lower end of the humerus, forming a hinge joint, whilst the lower end of the radius forms the greater part of the hinge joint at the wrist; the upper end of the radius and the lower end of the ulna enter into pivot joints by means of which the hand can be turned either palm upwards or downwards, the lower end of the radius revolving round the ulna.

Throughout the limbs the muscles are divided into two groups—the flexors, which bend joints, and the extensors, which straighten them. The chief muscles on the front of the forearm are flexors, and on the back extensors.

The hand is composed of eight wrist or carpal bones, arranged in two rows. From these spring the five metacarpal bones, to the ends of which the phalanges—three for each finger and two for the thumb—are attached. The five end phalanges are widened out to bear the nails. The spaces between the metacarpal bones are filled in with muscles and tendons, and so the palm of the hand is built up; the tendons of the muscles of the forearm run into the fingers, and are attached to the phalanges to move them.

The lower limbs are attached to the pelvis. There is a single bone in the thigh, the femur; its upper end is bent into a neck projecting inwards, and bearing the rounded articular surface that fits into the socket in the pelvis, forming with it a ball and socket joint.

The leg contains two bones, the large strong tibia inside with the fibula fastened to its outer side. The tibia joins in the hinge joints at the knee and ankle.

A patella, or knee-cap, is developed in the tendon at front of the knee.

Next comes the foot. The region of the ankle contains seven bones; the second largest (the astragalus) supports the tibia and rests on the largest (the os calcis), which forms the heel; to the latter the tendon of the muscles of the calf (the tendo Achilles) is attached. The muscles of the calf lift the heel from the ground in walking.

In the arch of the foot are five bones, the metatarsal, and in the toes the fourteen phalanges are arranged as in the fingers. The arched form of the foot gives springiness in walking.

It will be observed that the lower limb, although built on the same plan as the upper, differs from it in several respects. It is stronger because it has more weight to bear; the bones of the pelvis are immovably joined for greater strength; the hip joint is deep because support rather than extent of movement is required; there is a patella to give the muscles of the front of the thigh greater leverage to raise the leg in walking; the bones about the ankle are larger, stronger, and less movable than those of the wrist, and there are no pivot joints, as it would be useless to turn the sole of the foot upwards.

The bones of the limbs are liable to fracture; this may occur by direct violence, as the blow of a stick breaks an arm where it strikes it; or by indirect violence, when falling on the hand the ground stops the hand and the weight of the body is thrown against the inner end of the collar-bone where it joins the sternum; and a great crushing strain is put upon all the bones from the sternum to the hand, and the weakest is likely to give way. The collar-bone, which is curved like a long drawn-out S, or the radius where it is thinnest above the wrist are most likely to give way. It is this giving way at the weakest part that makes some fractures so common.

Fractures are divided into four classes:—

1. Simple, where the bone is broken, and no other serious injury is done; of course, in even a simple fracture some of the tissues about the break must be torn.

2. Compound, where the skin and tissues are torn, air reaching the fracture. This makes it much more dangerous and difficult to heal.

3. Comminuted, where the bone is broken into more than two pieces, as when crushed.

4. Complicated, where there is some other injury, as hæmorrhage, from the broken bone piercing an artery.

Handling a simple fracture carelessly may make it compound or even complicated.

The chief signs of fracture are: Pain, from injury to the nerves; distortion, which may be visible or felt by passing

Fig. 4.—Bones of the Foot.

the hand along the limb; shortening, from contraction of the muscles; loss of power, from want of the bone's support, and from the contracted state of the muscles; increased mobility when handled, the break forming, as it were, another joint; crepitus or crackling, when the broken ends grate against each other.

For the repair of a simple fracture the broken ends must be placed in position, and the limb supported by well-padded splints, firmly bandaged on, the joint above and below being kept at rest. First, the blood is absorbed; in about a week soft callus or repairing material is poured out. This forms a mass which surrounds and fixes the broken ends; this gradually hardens, and the part connecting the ends forms into bone. This process is complete in from six to eight weeks, but the lump of callus is only gradually absorbed, and can be felt for several months.

In compound fractures the wound must first be carefully cleaned and then treated so as to keep out any poisonous germs.

Dislocations, or displacement of the bones forming a joint also occur. The capsule of the joint and some of the ligaments will be torn, and so there is always bleeding under the skin.

A dislocation may be distinguished from a fracture. It occurs at a joint; there is no crepitus; there is both loss of power and lessened mobility when handled; the deformity is peculiar; the end of the bone may be felt in a wrong place. The limb is generally shortened in dislocation, as the muscles contract and draw the ends of the bones past each other.

To replace a dislocation the limb must be steadily pulled until the muscles are exhausted and relax; the end of the bone can then be replaced. Sometimes chloroform has to be administered to relax the muscles.

A sprain is a wrench or a twist; sometimes the end of the bone is pulled out of place and slips back again. The ligaments, tendons, &c., are strained and partly torn, and so blood is effused; this causes the blackness which gradually developes after a sprain, and it is the absorption of this blood and the repair of the torn tissues that makes recovery from a sprain so tedious.

### Great Exodus of Nurses to South Africa.

Without any fuss quite an army of nurses left Southampton for South Africa in the Union Castle steamer "Briton" on Saturday. The number (70) is the largest that has yet been sent in one vessel.

Nurses.—E. Andrews, L. Badger, G. Balfour, M. A. M. Borthwick, J. E. Bond, H. Burton, B. J. Betty, S. A. Boneham, S. A. Bailey, A. W. Barnett, B. E. Caws, J. M. Craig, M. Cross, B. M. Cornell, E. A. Couch, E. M. Denny, L. Dale, L. E. Dawson, A. F. Draper, E. Evans, A. Fincher, E. Fry, E. Fisher, M. G. Gilmore, M. A. Golby, E. M. Goold, E. P. Gibb, F. C. A. Holcroft, E. M. Hayden, E. H. Hay, M. A. E. Hopkins, A. A. S. Hill, C. M. Harvest, S. A. Kirkbride, L. G. Kinnear, V. J. Lamb, H. A. Lawrence, A. C. Lindsay, S. B. Lanyon, M. Little, S. Lamming, J. S. Lambe, M. O'C. McCreery, E. MacRae, C. M. Mello, G. A. E. Napier, G. Nutter, E. J. Northam, A. F. Plunkett, M. Perceval, A. A. Pallot, M. Roche, J. E. Rogers, M. Rae, A. M. Rose, M. E. Ransome, C. A. Shaw-Hellier, E. A. Snape, P. Schor, E. R. Sheriff, H. M. Shaw, A. T. Smith, M. Simey, M. Tonell, C. K. T. Taylor, C. Terry, K. Ward, G. Westbrook, M. Watson, and M. J. West.

The Training of Some of the Nurses.

Miss Lydia Badger was trained at the General Hospital, Birmingham, and has since August, 1892, been a private nurse on the staff of the Birmingham and Midland Counties Training Institute.

Miss Lizzie A. Bailey was trained at Leek Fever Hospital and Staffordshire General Infirmary. She was engaged as private nurse in connection with the Stoke-on-Trent Nurses' Institute for upwards of four years. Since April, 1897, she has been attached to the Nurses' Co-operation, and in the summer of that year was awarded the medal for nursing at the Maidstone typhoid epidemic.

Miss Josephine E. Bond was trained at St. Bartholomew's Hospital, and was staff nurse in the same institution for two years. She was sister at the East London Hospital for Children for three years, and since 1888 has been engaged in private nursing.

Miss J. M. Craig was trained at St. Bartholomew's Hospital, where she was afterwards staff nurse. From October, 1884, to 1894 she was attached to the London Association of Nurses, and has since then been engaged in private nursing.

Miss L. Dale was trained at the North-West London Hospital. She has had twelve months' experience in private nursing, and since 1898 has been charge nurse at the North-Western Fever Hospital, Hampstead.

Miss Edith P. Gibb was trained at Charing Cross Hospital for three years, and has been engaged in private nursing since 1896. She holds the massage certificate from the West End Hospital for Paralysis, Welbeck Street.

Miss Amy Agnes Sarah Hill was trained at the Free Hospital, Gray's Inn Road, for three and a half years. She has since been nurse at the South-Eastern Fever Hospital, the Royal London Ophthalmic Hospital, and in June, 1891, joined the Nurses' Co-operation.

Miss Edith Fry was trained at the London Hospital and has since been staff nurse at Stratford-on-Avon Hospital and Bucks General Hospital; night sister at the Royal Infirmary, Derby; temporary nurse at the Nursing Institute, Sheffield; sister at the Royal Albert Infirmary, Devonport; matron at the Cottage Hospital, Dartmouth; matron at the Miners' and Women's Hospital, Redruth; and matron since 1897 of the Cottage Hospital, Upper Norwood.

Miss A. M. E. Hopkins was trained at the North-West London Hospital. She has been private nurse for one year and for two years charge nurse at the North-Western Fever Hospital, Hampstead.

Miss Sarah Agnes Kirkbride was trained at the Royal Portsmouth Hospital for three years; was staff nurse at Lewisham Infirmary from November, 1895, to October, 1897; charge nurse at St. John's Hospital to June, 1898; and has been charge nurse since August, 1898, at the Infirmary, Vanbrugh Hill, Greenwich.

Miss Georgina Napier was trained at the Royal Hants County Hospital, of which she has also been sister. Her subsequent appointments have been at the Derby Royal Infirmary; a private surgical home for two years; matron of Charnwood Forest Convalescent Home; and, since August, 1898, assistant matron at the Royal South Hants Infirmary, Southampton.

Miss Ada Florence Plunkett was trained at Adelaide Hospital, Dublin, where she was subsequently staff nurse for two years and private nurse for two years. She has since belonged to the Nurses' Co-operation.

Miss M. E. Ransome was trained at St. Mary's Hospital.

Miss Caroline Augusta Shaw-Hellier was trained at Kimberley Hospital, South Africa, where she was staff nurse for upwards of two years. Subsequently she has been engaged in private nursing at Newport, Isle of Wight, and in connexion with the Royal Hospital, Bath. Since March, 1897, she has been occupied as a private nurse and masseuse.

Miss A. Lee Smith, of Sutton-on-Hull, was trained at the Royal South Hants Infirmary, Southampton. Since then she has been two years charge nurse at the General Hospital, Dover, and recently at the General Hospital, Croydon.

Miss E. A. Snape was trained at St. Bartholomew's Hospital, and since December, 1895, she has been sister at the City Fever Hospital, Leeds.

Miss Margaret Simey was trained at the London Hospital, and was staff nurse in the same institution for upwards of twelve months. She has been sister at the Hospital for Sick Children, Great Ormond Street, since August, 1896.

Miss Pauline Schor was trained at King's College Hospital for three years, and was afterwards staff nurse from November, 1890, to March, 1892. She has since been at the Dean Close Memorial School, and from 1894 to the present date a member of the Nurses' Co-operation.

Miss C. K. T. Taylor was trained at St. Mary's Hospital, and has since been sister.

### Nursing at Maritzburg.

### BY AN ARMY RESERVE NURSING SISTER.

A correspondent, writing from Maritzburg, says: We have had all sorts of experiences since our arrival. First, we were told on Monday, January 22nd, to be prepared to leave Cape Town at any moment and embark in the "Maine." That meant that we were to keep near the hotel. This mandate caused great excitement, and visions of being sent on to Ladysmith flashed before us.

Tuesday came, and we were just bubbling over, for we had heard we were to embark for certain on Wednesday. As we had a free afternoon we made up our minds to make the very most of it, so we went for a lovely drive.

Wednesday was rather unsettling. We packed, got ready, and at six p.m. started for the "Maine" in cabs by twos. On arriving at the docks we had to cross a coaling vessel, but even the discomfort of coal dust did not quell our ardour, or cause one single homeland sigh. We were pleased to meet Miss Hibbard, superintendent of the American nurses, and we had several little talks together on the way to Durban.

On the Sunday night there was a terrific hailstorm. They called it a "southerly buster." The hailstones were as big as small walnuts, and came down like bullets. Happily no one was hurt; part of the awning was torn right off, and a deck chair was blown away.

### Pleasure.

On Wednesday we landed at Durban. We took "rickshaws," and went in twos into the town and had a general survey all round, especially of the shops, until it was time to go to the station to see about the luggage. Five of the doctors and ourselves (eight sisters) met at the station at twelve p.m. to claim our luggage. This occupied over an hour, and then we all went and had lunch, and afterwards we went into a court belonging to the hotel and drank coffee, whilst three Indians came and showed snakes and did most wonderful tricks. Then the doctors left us, and we went for a tram ride. Fruit in Durban is very cheap, pine apples two or three pence each, very juicy and good, though small.

### Work.

At 5.40 p.m. we started for Pietermaritzburg in the train, and had a very pleasant journey. We arrived at 9.50, and were met by three army sisters. It was raining hard, and the streets were thick with mud, but we waded through nothing daunted, and arrived at a little house, where tea had been prepared for us. Subsequently we waded on to our huts. We each have a room—a long wooden shed with a door opening on to the verandah and a window next the door and one opposite. I am the lucky possessor of a cupboard, a small thing about one foot deep and three high; it has doors and one shelf, and I have several other shelves. The walls are just planks well put together. I have a double bed with a single mattress, one bolster, one pillow case, but no pillow (so I am doubly grateful for the nice one matron gave me), and two sheets. I have two wooden chairs. I am much better off than my neighbour, who has no cupboard and not many shelves. We have a basin each; her's stands on her chair and mine in the cupboard, and one can of water between us. I have no candlestick, and only a little bit of candle. Thursday morning we awoke very early. Sister's room and mine open into each other; there is no door between, so we talked and talked, and did not get up too early. We had breakfast in good time, after which we were sent back to wait in our quarters until the superintendent was ready to present us to the principal medical officer. In the waiting time I commenced this letter, but we were soon sent for and our wards were given to us.

### Nursing in the Fort.

Mine is in "the fort," a long, narrow, low stone shanty. The building forms three sides of a square with a large quadrangle in the middle. My first and principal ward contains 13 beds, which had been filled with convalescent surgical patients, bnt now contains enterics. On the opposite side of the quadrangle there are two small wards, one with four and the other with five beds, also under my care.

All the Army sisters here expect to be sent on to Ladysmith as soon as it is relieved, and we, of course, would like to be smuggled in among them.

I cannot be sufficiently thankful that we are not in tents. It rains all day and all night. Under existing circumstances one is glad because it is cool and there is no dust. But to lie in mud with pools and streams around, with rain dropping through the canvas, and without proper mackintosh beds—the very thought summons up a ghostly train of bodily ills, headed by King Rheumatism.

### BY AN ALEXANDRA NURSE.

February 21st.

This week we are rather in a jubilant frame of mind. Kimberley relieved, Cronje surrendered, and Ladysmith almost relieved. With friends close at hand we can afford to look a little brighter and to speak a little more cheerfully to each other. What a rejoicing there will be when Ladysmith is really opened up! Scarcely a family here but has some friend or relative shut up, and you can imagine with what glad expectancy they are looking forward to a reunion. But on the other hand many are as anxiously looking forward to this relief in order that they may go to visit the spot where their loved ones lie, shot down by shot or shell, or the victims of disease. One of my own women has just been widowed. Her husband, a sergeant in the gallant Dubs, was taken a prisoner to Pretoria, and there died from dysentery. His wife, a very superior woman, and five little children, are left to mourn his loss. This week some friends from town gave a concert to the wounded in camp. The programme was a first-class one; pianoforte and violin selections, with solo singing, formed an hour's delightful entertainment, and was much appreciated by the invalids. It was a verandah concert, and all the patients who could walk or be brought on wheel stretchers from the other wards were present, and showed their appreciation from time to time. The same friends had a few days previously given a large number of basket and deck chairs to the several wards for the use of the patients, and during an interval in the concert passed round cigars, cigarettes, matches, &c., to the Tommies. I wonder if you have any juvenile readers? If so, they may relish this. A little boy of four (son of a sergeant), who is ill at present, required a mustard plaster applied yesterday. On my putting it on he said, "Mamma, tell nurse I don't like curry down there; curry bites me." But he makes a very good little patient, and says, "I must be brave, for Daddy is a soldier."

March 1st.

This week was ushered in with glorious sunshine, and we could scarcely realise that fighting was being carried on with such deadly effects so near us till the wounded were sent down, 250 in number. Shells had done their very worst, and the poor wounded soldiers, officers and men alike, presented a most ghastly sight. Hearts and hands were taxed to the utmost, and it was far into the night ere all were seen to and comfortably disposed of. The death-rate has been rather high this week, some days no less than seven funerals taking place. Majuba Day brought the welcome news of Cronje's surrender. So there was joy and gladness in the camp, as well as outside. The Tommies knew that this had only been attained by the hard fighting of their brother soldiers on the other border, and at a terrible sacrifice of their lives, so it was with a subdued gladness this news was received.

The nurse whom I wrote of as having fever in Grey's Hospital is now almost convalescent, and is looking forward to resuming work shortly. As Ladysmith is now relieved our hospital accommodation will be taxed to the utmost. In preparation quite a number of civilian nurses were this week added to the staff; and, I suppose in honour of the relief of Ladysmith, to-day we had a group photographed of the nursing and medical staff. The city is jubilant, bunting, cheers, and music being seen and heard on every side. But there is another side to this picture, for very many are clad in mourning, and this very joyous display only tends to remind them of their recent loss.

### TRAVEL NOTES AND QUERIES.

The South for Health (North Briton).—Certainly, you may make any use of my letter that you like. I am very pleased to receive your kind appreciation; it is always so pleasant to know that one has been of real use. You are evidently a good traveller, and enjoy everything as it comes.

Ober Ammergau (Corinne).—You must book your places at once for the Passion Play, or you will be too late. You can do so through Dr. Lunn, or Messrs. Gaze or Cook. Dr. Lunn takes personally conducted parties on extremely moderate terms, and I should advise your going with one of these. I can send you a programme if you enclose to me a tamped addressed envelope.

### Nursing the Wounded at Naauwport.

By an Army Nursing Reserve Sister.

February 27th.—I came up country a few days ago to a newly-started hospital. We left Cape Town on a Saturday night and arrived at our destination at eight o'clock on the Monday morning. We were a party of five, and seats in two first-class corridor carriages had been reserved for us. As no one else got in we were very comfortable. We got breakfast at a station *en route*, also dinner, but that was not quite so satisfactory a meal. Luncheon and tea we had on the train as we had come well supplied with food. The nights were cold, and I was very glad of a Jæger sack and helmet to sleep in, feeling quite superior to my fellow sisters, who had only rugs. We arrived to find little brick, zinc-roofed, one-storied houses had been provided for us, which was luxury after being under canvas as I had been before. Across the road is our camp, which at present is not finished or settled in any way, though we are constantly getting sick and wounded down. However, I hope ere long it will be as settled and comfortable as other general hospitals, though nothing can make this spot nice. Sand—sand—sand, and nothing else to be seen except tents and travel-stained sunburnt up country to feel the reality of the war. I and one other sister are on night duty, and at present, as the hospital is soldiers in khaki. There are several regiments up here, and ten days or a week ago there was a Boer scare, but now the Boers are all supposed to have cleared off. Still, we are far enough only just opened, we are not so very busy. It is a most curious experience to wander round the marquees at night with one's lantern, and till tried you don't know how hard it is to make one's way through the sides of the tent; one pokes and pushes till one manages to manœuvre a way through. The officers' cottage is some way off, and we got utterly lost once going there to-night and had to get an orderly to assist us. Having only arrived to-day we do not yet know the lie of the land, and it is very puzzling work at first to find one's patients—every now and again one is challenged by a sentry and has to give the password. I know some night I shall forget to find out what it is. I never believed in the cold of African nights till my journey up here and till to-night, when we are frozen on our duty, notwithstanding thick cloaks. But the days are very hot. At the time I am writing this it is a quarter-past five a.m. and daylight has just come in.

March 5th.—We have been so busy since I began to write that I have not been able to add to this account of our doings. The hospital is nearly, if not quite, full, sick and wounded just poured in last week. There are the five enteric tents away from the others, with special orderlies; there also seems a good deal of dysentery about. There are some serious gunshot wounds. One poor fellow was shot through the lung, and another has a compound fracture of arm and leg; a third man is very badly injured in both hands and arms, but the majority of the wounds are not so very severe, and heal up well. The men are splendidly plucky and good; even amongst those just brought out of the train a great many are smiling and passing jokes. Some who came down last week had been travelling for nearly a week by bullock wagon and train. Poor fellows! they did look worn-out, dirty, and ill, but still ready to make the best of things, and delighted to be able to have a wash and sleep in a bed once more. They are all very keen to hear every bit of war news, and all are cheered up by the good reports lately. We have had some very bad nights. dimply wading through mud and water, an then trying to get into soaking tents is calculated to make one a little wet. I think one needs to be fairly strong to stand this work.

### Nursing in a Frontier Hospital at Bulawayo.

By a Special Correspondent.

Bulawayo itself is a howling wilderness—iron shanties scattered over a featureless plain. No trees, no views, no hills, hardly any flowers. As the most merciless of suns beats down upon all, the stray breezes that one sighs for only bring what are known locally as "dust devils"—columns which whirl with fiendish glee around the choking pedestrian. Streets exist only in name; at present they are foreshadowed by three houses in a line planted down innocent of plan or order. Outside the so-called city, however, there are villas built of bricks, and in these comfort, if not luxuries, abound in the matter of furniture and plate.

### My First Case.

I was packed off to a case in one of these domiciles the day after my arrival at Bulawayo. The family is one of the richest here, judging by the house and garden, the latter watered by windmills. All the walls are Minton-tiled, suggestive of coolness if nothing more. My patient's illness was trifling, but I nearly got sunstroke running up and down to the servants' quarters, to attend on coolie women in labour. My patient informed me that she usually confined her servants herself, the process being rarely of any but the simplest nature.

### The Private Hospital.

Upon my return to the private hospital to which I had engaged myself, I found I had to work in solid earnest. In addition to nursing I had all the pro.'s work of cleaning, bandage washing, tray-laying, and washing up, for there was only one Kaffir in the kitchen, the other one having run away because of our work. All the water and wood were fetched from a distance; the Kaffir has to chop the latter. The Chinaman in the kitchen, who should act as permanent cook, seems difficult to secure, or, at any rate, to keep when secured. If there is an excuse for the over-working of nurses and servants, it certainly is provided by the exorbitant prices of everything. The house, though small, is rented at £26 per month; living expenses correspond.

### My Present Work.

After a few weeks' work I was given an easier post, where I still am. At this hospital I am nursing in the native ward, where work is light. Three hours off duty, during which I roam about the extensive garden, in preference to roads that are either a waste of mud or dust. I have a charming strip of my own, which the recent rains have coaxed into a Paradise of lovely blossoms. While weeding I speculate on what is happening beyond the frontier. A perfectly withering censorship prevents any war news reaching us. This ignorance is often maddening. Meantime hospital work plods on, and thus far I have resisted all offers to exchange the monotony of the Kaffir wards for the over-work of the White ones. It will doubtless be wiser to stay where I am, for a change would only be to wards that are miserably "found" and woefully understaffed.

### The Condition of Nursing.

Imagine one nurse and two pros. for forty patients scattered over seven wards and endless corridors, for the hospital, like everything else in Bulawayo, is planned on a needlessly large and rambling plan. Off duty is hardly known, the good women employed over the white patients giving their time grudgingly to meals of a most comfortless description. The appliances for white and coloured wards are equally miserable. Fancy one set of bowls for every possible hospital need; nothing to distinguish them but dabs of paint, which the wretchedly incompetent pros. invariably disregard! Still, for a frontier hospital not five years old, and managed by trained nurses for only a year, expectations should not run high concerning methods of the strictly up to date character.

### The Doctor's Vigilance Needful.

The doctor here has just returned from a five months' visit to England. He is a most devoted student of the bacillus, and any nurse wishing to improve her acquaintance of the same has here ample opportunities of doing so. His vigilance in the wards is trying at first, but matter for admiration when one knows that it is a habit born of necessity, where Dutch nurses and pro.'s at least are concerned. It would make our sisters at home weep to see how life is thrown away through their carelessness and neglect. It is a common occurrence for them to give a typhoid suffering from hæmorrhage milk with cream floating thick on the top! Another instance of their methods is that of dragging a typhoid seized with vomiting into a half-upright position, in which unsupported condition he is expected to lean over the bowl! This sort of thing makes me very loath to take my off duty, and very inclined to adopt the carpet slipper methods of the doctor. A delightful feature in one's work is the arranging at times of lovely flowers sent by the Governor for the wards and the nurses' room. This week has brought a charming fern, quite new even in this land of varieties. I am told that it grows in abundance somewhere out on the Matopo Hills, where streams we thirst for here trickle down the slopes.

### Kimberley Experiences.

Yesterday I took relief duty in the White convalescent ward, where I made the acquaintance of a nurse who had spent five months in the mining hospital at Kimberley. She gives her experience as that of simple cases, requiring little beyond a few medicines and small dressings. It was interesting to hear what she said of the working side of the Kimberley young men. I had seen the recreative one many times in Cape Town, when the influx of "Kimberley Boys" who arrive with the earnings of months to spend is something to see and remember!

### The Climate.

A distinct gain in Bulawayo over the Colony is the absence of the south-easters, and if only water were more plentiful, the climate on the whole is distinctly bearable. A point to accentuate, however, is the irritability that the climate either develops or sets up in everybody. Merriman, the author, alludes to a microbe—the Irritability of South Africa—and I am bound to admit the truth of his little pleasantry. In normal times the outlook for nurses in Bulawayo anxious to work on their own account is fairly promising, but, of course, now is not the time to think of such a venture.

### Novelties for Nurses.

### THE QUAKER BATH CABINET.

The Quaker Bath Cabinet is a simple and effective and inexpensive contrivance whereby the benefits of a Turkish or vapour bath can be obtained in the home. The bath consists of a fourfold screen of wire and American cloth closing at the top, with an opening only large enough to permit the head of the bather to remain outside. Inside the bath a chair is placed, and underneath stands the lamp which provides the heat for the hot air or the vapour. In cases of colds or rheumatism the bath will be found a great comfort, and under medical advice there are many ailments in which it might be usefully employed. Full directions are supplied with the bath, which reveal the numerous uses to which it can be put. The contrivance is both light and portable.

### The Commissariat Department.

By HELEN TODD, Matron of the National Sanatorium, Bournemouth.

### II.—BACON, BREAD, MILK PUDDINGS, &c.

LAST week I dealt with beef and mutton and the loss of weight in cooking, which I put at 30 and 50 per cent. respectively. The next article of food on the list is bacon, which is chiefly given as a breakfast dish. It is a particularly useful article in the dietary of the phthisical patient on account of its large proportion of fat. One naturally expects to find a greater loss in frying than in boiling bacon, and this is fully justified by figures.

After frying 12 lb. of bacon, I find it (including its dripping which can be eaten) to weigh only 9 lb., so that the loss has been just 3lb., or 25 per cent.; a piece the same size boiled would lose only $9\frac{3}{13}$ per cent. It is somewhat surprising at first to compare this with the loss of weight in cooking beef and mutton. Leaving all bone out of calculations the loss in cooking is $32\frac{9}{13}$ per cent. of an average-sized leg of mutton; $28\frac{4}{7}$ per cent. of beef, but only 25 per cent. of the fried bacon.

### HAM AND TRIPE.

Boiled hams are, next to mutton, the most extravagant form of meat that has come under notice. This is due to the great length of time required for its cooking. Taking a ham weighing, when raw, $11\frac{1}{4}$ lb., I find the tough skin to weigh $\frac{3}{4}$ lb., and the bone, when scraped, 1 lb.; the loss of weight in the long boiling amounts to 3 lb. 4 oz., so that only $6\frac{1}{4}$ lb. are left for dietary purposes out of our $11\frac{1}{4}$ lb., showing a total loss of $40\frac{4}{9}$ per cent. of the original weight of the ham.

Tripe, for some unexplained reason, is rarely an institution dietary, and yet it is most useful, having the great merits of digestibility and economy, whilst the working classes, from whom our hospital patients are chiefly drawn, are, as a rule, exceedingly fond of it. It is especially suitable for phthisical patients, and a much appreciated addition to the five o'clock meal, making a pleasant variation from eggs, ham, or marmalade. Being stewed in milk it is also valuable as a fresh vehicle for that indispensable article of the consumptive's diet.

### THE STAFF OF LIFE.

From the consideration of meat one turns as a matter of course to that of bread, the "staff of life." Here it will be found that variation of weight is not only caused in the cooking, but is also materially affected by the length of time the bread is kept before it is used and the manner of serving.

According to the three dietaries already mentioned, the allowance per head per diem in the general hospital for a man on full diet is 16 oz., and for a woman 14 oz. The Poor Law children have $13\frac{1}{2}$ oz., and the sanatorium patient 20 oz. Bread for institutions is generally sold in long 4 lb. loaves, two of which are reckoned as a gallon. The dough is weighed before baking, and a certain allowance made for inevitable loss of weight in that process; the loss, however, varies, and it is therefore necessary to weigh the bread in the bulk as it is delivered by the baker, that any loss may be noted and made good by him. From the daily record kept in this institution I find the average loss requiring to be made good on every 28 lb. is 1 lb. Bread, if kept for any length of time, rapidly loses weight—a 4 lb. loaf loses on an average 2 to 3 oz. in 24 hours, and 4 to 6 oz. in 48 hours. In ordering supplies, therefore, the fact that the bread will be delivered and consequently weighed when "new" must be taken into account, or 24 hours later the required weight will not be forthcoming.

### "NEW" BREAD.

If bread be cut and served straight from the bakehouse it will not only be indigestible for the great majority of patients, but there will also be a large amount of waste from crumbling in the cutting, which can be avoided if it be stored for 24 hours before using. This point was especially brought home to the authorities of a certain Poor Law institution where the bread was made and baked on the premises, and rarely stood for any length of time before being served, when it was cut into slices with an ordinary bread machine. It was found that the loss in that cutting are enormous; bread that scaled 155 lb. when in loaves, on cutting weighed 146 lb., showing a loss of 9 lb., or $5\frac{25}{31}$ per cent.; 146 lb. being the average daily consumption, it will at once be seen how excessive was the waste. The superintendent was determined to reduce this, and had the experiment made of baking the bread in buns of 3 oz. and 6 oz. weights, which only needed to be separated, or, at most, dividing into two before serving, and the loss was reduced from $5\frac{25}{31}$ to $\frac{20}{31}$ (*i e.* from 9 lb to 1 lb.) per cent., and 8 lb. of bread a day were saved—2,920 lb. in a year.

### BUTTER.

Butter is not an item of hospital diet in which there should be any waste; the hospital scale of $\frac{1}{2}$ oz. per head per diem does not err on the side of extravagance, as every unfortunate probationer who has striven to make it butter the patients' bread for breakfast, lunch, tea, and supper knows only too well. The Poor Law child comes off better with $1\frac{1}{2}$ oz. of butter or margarine daily, whilst the sanatorium patient proudly heads the list with his allowance of 3 to 4 oz. a day. Fresh butter is more economical than salt, containing as it does less water, and the *weight* of the article being entirely fatty food instead of the heavy salts.

### MILK PUDDINGS.

Milk puddings form a very necessary and prominent feature in invalid cookery, the quantity given varying, of course, in an inverse ratio with the more solid articles of food on the diet scale; a man on "full diet" in a general hospital having an allowance of 4 oz., whilst one on "convalescent diet" has 8 oz. The Poor Law school children are given 7 oz. A sanatorium patient can rarely manage more than 8 oz.

Rice is the favourite grain, but in hospital economy it is by no means the cheapest. The proper proportions for the different weights of the various kinds of grain used in making milk puddings are: For puddings of 16 oz. weight, rice, $2\frac{1}{2}$ oz.; sago, tapioca, barley, or semolina, $2\frac{1}{4}$ oz.; macaroni, or hominy, 2 oz. The other ingredients never vary, being $\frac{3}{4}$ oz. of sugar and 1 pint of milk in each case. In buying for large institutions grain is generally obtained by the cwt., and according to the above table of quantities

| | | | | | |
|---|---|---|---|---|---|
| Rice at | 17s. | per cwt. | would make | $716\frac{4}{5}$ | lb. puddings. |
| Sago at | 17s. | ,, | ,, | $796\frac{4}{9}$ | ,, |
| Tapioca at | 16s. | ,, | ,, | $796\frac{4}{9}$ | ,, |
| Barley at | 15s. 4d. | ,, | ,, | $796\frac{4}{9}$ | ,, |
| Semolina at | 19s. | ,, | ,, | $796\frac{4}{9}$ | ,, |
| Macaroni at | 24s. | ,, | ,, | 846 | ,, |
| Hominy at | 22s. | ,, | ,, | 846 | ,, |

(These prices, of course, vary a little from time to time, but may be taken as very fair average prices.)

### THE COMPOSITION OF MILK PUDDINGS.

These puddings are chiefly useful as vehicles for milk, and having compared the relative prices and weights of the various grain, it is instructive to glance at the composition of the puddings themselves, and see what is the percentage of milk and grain in each.

In a rice pudding we have—

| | | | | |
|---|---|---|---|---|
| Rice | ... | $15\frac{10}{16}$ | per cent. | of the whole. |
| Milk | ... | $79\frac{11}{16}$ | ,, | ,, |
| Sugar | ... | $4\frac{11}{16}$ | ,, | ,, |

Compare these figures with those relating to sago, tapioca, barley, or semolina, where we get—

| | | |
|---|---|---|
| Grain | ... ... ... | $14\frac{4}{16}$ per cent. |
| Milk | ... ... ... | $81\frac{3}{16}$ ,, |
| Sugar | ... ... ... | $4\frac{9}{16}$ ,, |

The loss of weight in cooking these puddings is extremely small, a pudding of 7½ lbs. losing on an average 1 oz. during the process. If, however, it is allowed to be given cold, as is frequently the case in very hot weather, the same sized pudding will lose 1 lb. in 24 hours. The size quoted will be found the most convenient for institution use, a much larger size being both awkward to cook and handle. Puddings are one of few articles of diet in which there is practically no waste.

Porridge is a "grain food" which is exceedingly useful in invalid dietary; being an additional milk vehicle and a weight-forming food, it is especially useful for phthisical patients. A gallon of porridge, which will serve 16 persons with half a pint each, consists of 32 oz. of oatmeal and 8 pints of water at a cost of $2\frac{4}{7}$d. The oatmeal known as "medium" is the best for sick persons. If procured direct from the mills in Scotland it can be had for 12s. per cwt., and is in every respect better than that sold in this country.

### Jottings on Hospital Work in the United States.

By a Matron.

In many ways this hospital, of which I have now been matron some months, is very nice, and the buildings are extremely good, though some of the arrangements seem to my English ideas very peculiar, and there is a great deal of superintendence necessary, especially amongst the servants—all of them black, of course, with woolly hair. Fortunately, the cook is very good, and besides her there are two laundresses, two ward-maids, a butler, and a porter. But they all require absolutely driving, and at no time will they work with any zest or interest, being lazy, indolent, and procrastinating. One has to be constantly running round after them, which is a great nuisance; and I am afraid I am sometimes rather too lenient; then things get desperate and I have a fling all round.

There are nine nurses—all Americans except two English. The American probationers, speaking from my own experience, are very independent, and think it is terribly hard lines to have any cleaning or dirty work to do, being so accustomed to have it all done by the "darkies." I have already had a great many applications for two probationer vacancies, but nearly all from girls in the town, which are not at all acceptable. There is a great deal of surgical work here—our patients are all women—the operations are done very quickly and wonderfully neatly. A good many paying patients are received, and some private nursing is done, but this does not amount to much, though it has caused more bother and trouble than any of the other work. At first the committee insisted upon Nurse E——, who came out with me, going out private nursing and doing all kinds of work; not, in fact, being a charge nurse or sister at all, as previously arranged. But the matter was at last amicably settled, and she now has charge of the children's ward of six beds and the private patients. Though I like America very much, and have never once regretted that I came, I am sure I shall not be able to afford to come home for a holiday—everything here is more expensive, and I find the clothing I brought with me not at all suitable, the climate being so much hotter than I expected, though it is colder sometimes, but only for a few days at a time.

### Appointments.

Tamworth Cottage Hospital.—Miss Mary Anne Clark has been appointed Matron. She was trained at Tamworth Cottage Hospital, and has since been engaged at the York Home for Nurses for three years, charge nurse at Birmingham and Midland Eye Hospital for one and a half years, and head nurse at Tamworth Cottage Hospital for three years.

Bath Eye Infirmary.—Miss Helen Hadley has been appointed Matron. She was trained at the Royal Eye Hospital, Southwark, and London Temperance Hospital, and has since been nurse at the Manchester Eye Hospital and the London Throat Hospital.

Hastings Hospital.—Miss Jessie Barber has been appointed Matron. She was trained at St. Thomas's Hospital, London, and has since been assistant matron at the County Asylum, Chartham Down, matron at Folkestone Hospital, and assistant matron at Birmingham Infirmary.

Wolverhampton and Staffordshire General Hospital.—Miss Ida M. Eastwood has been appointed Matron. She was trained at the London Hospital, and has since been matron at Brentwood Cottage Hospital for upwards of four years, and assistant matron at Bristol Royal Infirmary.

Hertford General Infirmary.—Miss Alice A. Smith has been appointed Matron. She was trained at the London Hospital, and was subsequently sister. She has also been night sister and home sister (for two years) at the Poplar Hospital for Accidents.

Tunbridge Wells General Hospital.—Miss L. P. Lessey has been appointed Matron. She was trained at the London Hospital, and she has since been matron at Boston Hospital, lady superintendent to the Taunton and Somerset Hospital, and Victoria Nursing Institute, Taunton.

Poplar and Stepney Sick Asylum.—The name of the new Senior Assistant Matron is Butler, not Buller.

### Minor Appointments.

Plymouth Workhouse Infirmary.—Miss Florence Thompson has been appointed Assistant Nurse. She was trained by the Meath Workhouse Attendants' Association at St. Luke's Hospital, Halifax.—Miss Rose Price has been appointed Assistant Nurse. She was trained by the Meath Workhouse Attendants' Association at the General Hospital, Cheltenham.

City of London Hospital for Diseases of the Chest.—Miss Emily Griffiths has been appointed Staff Nurse. She was trained at Crumpsall Infirmary, has done private nursing for two years, and for the last two years has been nurse at the Park Fever Hospital.

The Officers' Hospital, Murree, India.—Miss M. MacMunn has been appointed Sister by Lady Roberts. She was trained at the Charing Cross Hospital, and for some years had charge of the Alexander Ward. She has since been working as sister in the Convalescent Hospital at Ilkley.

Nantwich Workhouse Hospital.—Miss Alice Gardner Dibblin has been appointed Superintendent Nurse. She was trained at Woolwich Workhouse Infirmary, Plumstead, and has since been night superintendent and nurse in the same institution.

Bury Union Isolation Hospital.—Miss Mary Davies has been appointed Charge Nurse. She was trained at Merthyr Tydfil Union Infirmary, and holds the L.O.S. certificate.

### Echoes from the Outside World.

### AN OPEN LETTER TO A HOSPITAL NURSE.

There is still little news from the scene of operations in South Africa. Colonel Baden-Powell continues to hold out, and as yet there seems only a slight prospect of his immediate relief, for Colonel Plumer appears to be unable to force his way through the Boers. Help is expected to come from another source. General French has returned from Thabanchu, and Lord Kitchener is rejoining Lord Roberts, so that there is every probability of an early advance being made, though till all transport arrangements are in working order, and the safety of the railway is assured, the commanders are not likely to make a start. The rebels go on giving up their arms to the English, but they are frequently of such an antiquated pattern that serious doubts are entertained whether they are the same weapons that were used in the struggle, and whether the owners have not quietly buried their others, awaiting further developments. The Dutch who have been loyal to us all through the troublous times complain that it is not fair these men should be allowed to return to their homes and have no punishment whatever, but be placed on a level with those who have been staunch and true. A proposal has been made that the exigencies of the case would be met if the rebels were disfranchised for a certain number of years, and the suggestion seems a good one. In this, as in other matters, entire confidence may be reposed in the judgment of Lord Roberts. As I write, a report of the death of General Joubert is prevalent, and seems to be well founded.

The mail letters from South Africa this week make delightful reading, for the news of Cronje's surrender and the relief of Ladysmith had just been received, and the joy and gladness was so great that, to quote the words of my correspondent, "Durban was delirious." Success, in a way, means more to the residents in Natal than to us here, because their numerous relatives at the front have, in most cases, gone as volunteers for the war in Natal only, and will no longer be called upon to fight as soon as the Boers are driven back over the border. Such, however, is the military spirit amongst the Colonials that those who are unmarried or unemployed will probably ask to be allowed to accompany the regulars into the enemy's country. Another result of the splendid strategy of Lord Roberts and Lord Kitchener is the effect which has been produced upon the natives. "It is only now," says my informant, "that we are realising the doubt which has existed in the minds of the natives as to our success, and we cannot help admiring the quiet way they have kept their own counsel and secretly made their plans without asking questions or discussing events with us. The triumphs of the last few weeks will leave a lasting mark upon one little girl, for I learn that a baby born on March 1st is to be christened 'Roberta Cronjina Majuba.'" Poor wee chick! Whatever will she be called? "Bobs," I suppose.

We women in England can do little for those at the war except send them money and comforts, our wishes and our prayers. Nurses at the front are more fortunate, and so are two women I have heard of lately. One is the wife of a man who is in command at a small railway station through which the soldiers all pass on their way up country from Durban. This kind soul, although she has no large staff of servants—only station coolies and a refugee to help—has given tea to more than forty thousand soldiers and officers since the war began. For the latter the meal is sometimes put in the waiting-room, but as time is an object, and the soldiers are often numerous, as soon as the train is drawn up men, women, and children, all armed with a teapot or a jug, are ready to empty the welcome beverage into the canteens of the "Tommies." Occasionally the troops only have their water-bottles with them, into which it is slow work to pour tea. Then often the jug is seized and a good pull is taken. Biscuits, cakes, and now and then fruit are also handed round. How proud that stationmaster must be of his wife! The work of the other good Samaritan is quite different in character. At Harrismith there are a large number of English prisoners, and these poor fellows, looking sadly down-hearted, are allowed to exercise each day in a certain part of the enclosed square. A lady, whose husband lives in the square, used to watch the melancholy procession with aching heart, till one day she had the brilliant idea that perhaps they would like her to sing to them. So she now daily looks for the arrival of the dreary little band, and directly they appear she opens wide all her windows, and singing with all her strength, that the words may reach them, she goes through as many as possible of the English and Scotch ballads. The sweet sympathy must be very welcome to the captives, and will do much to remind them that, though absent, they cannot be forgotten by loving ones at home, when even a stranger will take so much trouble on their behalf.

Just now when the question of better homes for working women is so much to the fore, I think the account of a visit paid to one of the Rowton Houses by a "lodger" may be worth repeating. My informant, a well-to-do landowner, who takes much interest in the subject of the comfortable housing of his own labourers, determined to see for himself how the scheme was worked, and accordingly one evening he applied for shelter for the night at the King's Cross house. This was between half-past five and six o'clock in the evening, when he formed one of a long queue. At last his turn came, but so near the end that he began to fear lest he should lose his opportunity. However, he was given the coveted ticket in exchange for sixpence, and told that as he had now secured his bed, he was at liberty if he wished to go out again. He did so, but returned early enough to have a chat with several of his fellow lodgers. He found that a large number were gentlemen or semi-gentlemen, who had come down in the world, though there were many working men, who were regular tenants of their cubicles, evidently fancying themselves rather swells in consequence. One man said, "It's a slap-up place, but rather expensive you know—3s. 6d. a week." Each cubicle has only one occupant, and is lighted from outside, no candles or lights being allowed inside. Washing and dressing is done downstairs in a lavatory with eighty basins, and my friend, who slept comfortably, had breakfast before sauntering forth—bread and butter and tea and two kippers for sixpence—and considered that he had excellent value for his total expenditure of one shilling.

Two attempts have been made recently to protect the children of the present generation from evil habits. In the House of Commons a Bill to render the sale of intoxicants to children under 16 years of age illegal has reached the Committee stage. The other is a local effort. The Beckenham School Board Management Committee having requested one of their medical officers to draft a circular for distribution among the parents explaining the injury caused to children by cigarette smoking, this has been done, and the circular will soon be widely circulated. It shows that smoking by boys impairs eyesight, upsets the nerves, disturbs the digestive organs, and stunts growth. I imagine, though, that in many cases the habit is deprecated as much by the parents as by the School Board, but the young imps smoke on the sly. The only way to bring about a good result is to appeal to the children direct. A few may see for themselves the wisdom of deferring their smoking days, but a system of rewards for abstainers will, I fancy, be necessary to keep the youths in the way they should go.

### A Book and its Story.

### THE MAN OF THE HOUR.*

MR. JERROLD's concise and handy volume, made accessible to the general reader by its modest price, contains all the leading incidents in the military career of Field-Marshal Lord Roberts. Born in 1832 at Cawnpore, he is the soldier son of a soldier father, who reaped his laurels also in India, the country to which General Roberts has given the best years of his life. General Sir Abraham Roberts, K.C.B., G.C.B., retired from the East India Company's service in 1853, leaving his son to maintain the distinguished reputation which he had gained by devotion to the service of which he was a loyal and illustrious officer.

Lord Roberts is, like the Duke of Wellington, an Irishman. From both parents he comes of Irish descent, with a strain of French Huguenot blood on his mother's side. The city of Waterford claims the distinction of being the birthplace of his father, and also of previous generations of the family. His early years were spent, as those of all Anglo-Indian children are, away from his parents. At Clifton his childhood was passed, and from there he entered Eton, the public school from which so many famous soldiers and statesmen have gone forth, Sir Redvers Buller among the former. Two years later his military education commenced at Sandhurst. From Sandhurst he went to Addiscombe, and from that college passed out into the Indian service. In spite of not over-robust health, he stood ninth on the list of four dozen successful candidates, shortly afterwards receiving his commission as second-lieutenant in the Bengal Artillery.

In February, 1852, the young soldier sailed for India. His journey was one "destined to be significant of much, not only in the life-story of the soldier himself, but in the history of his country. . . . India really represented to the young cadet much that is most significant in the word "Home"—it was his birthplace, and it was there that his father was holding an honourable command after half a century spent in the military service of the growing Indian Empire."

It was to "Dum-Dum," the place whose name is familiar to everyone just now in connection with "the deadliest bullets used in modern warfare," that Frederick Roberts was appointed on his arrival in India. In August of the same year he joined his father at Peshawar as aide-de-camp. "Major-General Roberts, who was then in his sixty-ninth year, had seen much of Afghan warfare in the troubles of a decade or so earlier, and his knowledge and advice must have stood his son in good stead when a quarter of a century later, he was performing his notable services in the same part of Asia." This year saw the retirement of his father "after half a century of sterling service in India." Six months later he was appointed lieutenant in the Bengal Horse Artillery, a force described by Colonel Mallerson as "unsurpassed and unsurpassable," and one which he wished to join, since he fell in with a body of it, soon after his arrival in the country.

The clouds which were gathering broke later in the storm of disaffection known to us as the Indian Mutiny. It was on the eve of that terrible time that young Roberts received, in addition to his new commission, an appointment which is noted as the "turning point in his career." He became, when at Simla, acquainted with Colonel Becher, Quartermaster-General, and he, recognising instinctively that here was a man of no ordinary parts, desired, should occasion arise, to have him attached to his staff. At that time Roberts' knowledge of Hindustani was not up to the necessary standard for officers in that department. Strenuous application on his part, however, soon enabled him to pass the test examination. A temporary appointment was then given on condition that he "doubled his part," which meant that in addition to performing regimental duties he would have to attend also to those incidental to his new post. Thus "he got his foot on the first rung of the ladder of promotion." At no time a man of robust physique, he has made up for it throughout life in a marvellous manner by strength of will, indomitable energy, and great mental activity. There is more than one reference to absence on "sick leave" necessitated by unflinching attention to arduous duties bravely undertaken at all hazards. As an instance of his energy and devotion to duty we read, that on relinquishing his temporary appointment, spoken of above, of D.A.Q.M.G., it was necessary for him to join the General of Division at Rawal Pindi. He desired to stay with troop to the last moment. Starting early in the morning, he rode one hundred miles with only a short rest in eleven hours, and joined General Becher the same evening. Three months later Lieutenant Roberts joined General Barnard's small force, encamped on the ridge overlooking Delhi. He was wounded in one of the numerous fights which took place before the city was recaptured, his life being saved by a leather pouch, usually worn in front, having slipped to the back, which, breaking the force of the bullet, prevented it penetrating any distance.

Hodson, of Hodson's Horse a name inseparably associated with the Indian Mutiny, records at the close of the day's engagement, "Chamberlain shot through the arm, and little Roberts." Throughout the thrilling scenes of the Mutiny Lieutenant Roberts was actively engaged. He bore a charmed life, and had many narrow escapes, being told off on more than one occasion for hazardous duties, which he performed at great personal risk. Any notice of his life would be incomplete without reference to the special deed by which he gained his V.C. It was on New Year's Day, 1858, that, when engaged in the pursuit of the enemy who were in retreat, Lieutenant Roberts, riding by the side of a fellow-officer, saw him fall; as he was turning to his assistance he saw a native soldier in deadly peril from a rebel, who was about to bayonet him. He at once galloped to his assistance, and cut down his opponent. Two Sepoys at a distance were now making off with the standard. He cut them down and rescued the colours, having a miraculous escape, for a musket was fired at him as he rode away, but it missed aim, and plucky "little Roberts" rode back in triumph with the standard. There is a pathos attached to the gaining of this coveted distinction by our hero's son, some forty years later. He, as we know, died from the wounds received in his gallant attempt to save the guns at Colenso in the present war, and expired on the day that he received the honour. We feel sure that our readers will find much to interest them in this readable little book, which has some good plates—one, a portrait of Lord Roberts, and another, a family group of Lady Roberts and her son, and one daughter.

* "Lord Roberts of Kandahar, V.C. The Life Story of a Great Soldier." By Walter Jerrold. (Publishers, Partridge and Co., London. 2s. 6d. net.)

### To Nurses.

WE invite contributions from any of our readers, and shall be glad to pay for "Notes on News from the Nursing World," or for articles describing nursing experiences, or dealing with any nursing question from an original point of view. The minimum payment for contributions is 5s., but we welcome interesting contributions of a column, or a page, in length. It may be added that notices of entertainments, presentations, and deaths are not paid for, but, of course, we are always glad to receive them. All rejected manuscripts are returned in due course, and all payments for manuscripts used are made as early as possible at the beginning of each quarter.

### Everybody's Opinion.

[Correspondence on all subjects is invited, but we cannot in any way be responsible for the opinions expressed by our correspondents. No communication can be entertained if the name and address of the correspondent is not given, as a guarantee of good faith but not necessarily for publication, or unless one side of the paper only is written on.]

### INFANTILE PARALYSIS.

"Nurse Stout" writes: I should be so glad to know if any of the readers of The Hospital have ever had a case like this? A baby eight months old was seized with infantile paralysis; a bad discharge comes from the ears, and now a lump has suddenly appeared on the side of the head which seems to be getting bigger. Is it likely to have anything to do with the above ailments? Any information will much interest Nurse S. on the subject.

*** Show this to the doctor at once.—Ed. *T. H.*

### THE RECORDING CLOCK.

"A Late Mental Nurse" writes: Does "A Well-Wisher of Hospitals" mean to suggest that asylum workers as a class are looked upon as not fit to be trusted? or does she mean that the use of the clock in her hospital classes her among individual members of the various branches of the nursing profession, including mental nursing, who are not fit to be trusted? I would also say that noiseless recording clocks are at present extant.

"Tickers" writes: In answer to a "Well-wisher of Hospitals," I should like to say that recording clocks—or, as we call them, "tell tale clocks"—are in general use in St. Luke's Workhouse, City Road. I am on night duty myself, and have two clocks to peg every hour, one in each of my wards. They make no more noise than an ordinary clock ticking. They are small wooden cases fixed on the wall, with a wheel inside round which is a tape with hours, half-hours, and quarters marked on it. A peg arrangement is on top of the clock, and this is pressed every hour and makes a pin prick on the tape. It is wound up every day, the tape renewed, and the little door of the clock is locked. There is a small glass face in the door of the clock just large enough to show the hour. I quite agree with "A Well-wisher of Hospitals" in thinking that night nurses should be trusted without these clocks. At the same time, if "A Well-wisher of Hospitals" has ever nursed the class of patients one meets with here or in other large workhouses she would be very glad at times that she had a "tell-tale clock." It has often been said by objectionable patients that night nurses do not go round their wards as often as they should, so that in cases of doubt, &c., it can be proved conclusively by the clock to the satisfaction of the authorities that the night nurse is doing her duty in that respect.

### A WANT.

"Sister Alice" writes: In most hospitals the nursing staff is considerably less at night than during the day, also in a great many hospitals "pros." are put on night duty. Why this should be is strange, because at night especially medical, but also surgical cases, require quite if not more attention. Then again, surely the most experienced should be left in charge at a time when sisters and matron are absent and the doctors not so readily to be got at. Among other improvements, perhaps, some hospital may start an entire staff of night nurses corresponding to what is required during the day. Of course it will be said that a great deal of manual work is done during the day, but how can a nurse, no matter how competent she may be, do her duty when she is left in charge of thirty-five or thirty-eight surgical cases (women and children), especially after an operation day? I myself have had two women in different wards vomiting after an anæsthetic, a little girl calling for a drink of water (burns), and another patient wanting attention. In such cases the work cannot be done properly, and who suffers? The unfortunate patient—a suffering that need never occur if even a few more nurses were put on night duty in our large hospitals. This is a want our trained matrons can remedy themselves.

### A WARNING.

"Nurse M." writes: On February 17th I had an advertisement in The Hospital seeking an engagement. A gentle and refined lady called upon me in response. She wished to see me in preference to writing. She wanted me to nurse her brother. She engaged me, and I was to go to Bexley Heath the next day (Saturday). In order to pay her railway fare back the lady put her hand into her pocket and found that her purse was gone. She said she did not know how she should get home, and, as she had several purchases to make, asked if I could lend her 30s. I told her I could not, for I had been ill with pleurisy for several weeks. Then she said could I borrow it; I should be sure to have it the next day. I borrowed it of a friend, and when I went to Bexley Heath there was no Madame Aldridge at "the Cedars." The police told me they have wanted a person answering the description for some time. Some of my patients wished me to write this.

### COMPULSORY HOLIDAYS.

"Justice" writes: Could any of your readers kindly set me right on the following legal matter? The hospital to which I am attached has been closed for some weeks, and the staff requested to take their summer holidays during the time. Would it not be right for the nurses to claim board-wages from the period of the expiration of their holiday? The closing of the hospital was quite unlooked for, and we think that some compensation is due to us. Personally, I have had to come abroad, which I should not have done had the holidays been at the usual time, and have now, besides the expense of the journey, hotel bills to pay for. Several of the other sisters have been put to a like expense, also the probationers. We do not contest the fact of having the holidays now,as we did not agree for any specified time; but after the holiday month we think the committee ought topay us board-wages. Can anyone help us with a reliable opinion?

### Presentations.

Tunbridge Wells General Hospital.—Miss F. L. Hay Forbes, having resigned her post as matron of the Tunbridge Wells General Hospital, has been presented by the nursing staff with a very handsome gold curb bracelet, with padlock, as a token of the esteem and affection with which they regarded her. The domestic staff gave her a pair of hand-painted Worcester china dessert dishes. Miss Forbes was likewise the recipient of many other gifts from various friends, amongst which may be mentioned a pair of silver flower vases, all of which were presented with expressions of affectionate regard, and of sorrow at her leaving the post she had held for so long.

North Bierley Joint Hospital.—Miss Edith Evans has been presented by the nursing staff with a fitted writing desk in crocodile leather, with silver mounting, to mark the completion of her term of probationership and promotion to the rank of staff nurse. Dr. Sutherland, the medical superintendent, in making the presentation, spoke in expressive terms of the conduct and efficiency of Nurse Evans, who, in acknowledging the gift, said she wished to take the opportunity of thanking her instructors and expressing her gratitude for all kindnesses shown by the doctor and matron.

Chorlton Union.—The nursing staff of Chorlton Union have presented their late senior resident medical officer (Dr. Kelsall) with a handsome silver cigar case and match box on his departure for the front. Dr. Kelsall will be very much missed both by nurses and patients, as he was extremely kind and courteous to all.

### For Reading to the Sick.

"My grace is sufficient for thee; My strength is made perfect in weakness."

Teach me to live! 'Tis easier far to die—
Gently and silently to pass away—
On earth's long night to close the heavy eye
And waken in the glorious realms of day.

Teach me that harder lesson—how to live,
To serve Thee in the darkest paths of life;
Arm me for conflict—now, fresh vigour give,
And make me more than conqueror in the strife.

Teach me to live, Thy purpose to fulfil;
Bright for Thy glory let my taper shine;
Each day renew, remould this stubborn will,
Closer round Thee my heart's affections twine.

Oh! look not after great things; small breathings, small desires after the Lord, if true and pure, are sweet beginnings of life. Take heed of despising "the day of small things," by looking after some great visitation, proportionable to thy distress, according to thy eye. Nay, thou must become a child; thou must lose thy own will quite by degrees. Thou must wait for life to be measured out by the Father, and be content with what proportion, and at what time, He shall please to measure.—*I. Penington.*

It is possible, when the future is dim, when our depressed faculties can form no bright ideas of the perfection and happiness of a better world—it is possible still to cling to the conviction of God's merciful purpose towards His creatures, of His parental goodness even in suffering; still to feel that the path of duty, though trodden with a heavy heart, leads to peace; still to be true to conscience; still to do our work, to resist temptation, to be useful, though with diminished energy; to give up our wills when we cannot rejoice under God's mysterious providence. In this patient, though uncheered obedience, we become prepared for light. The soul gathers force.—*Wm. E. Channing.*

There is one favour which God confers in this life on the soul which has placed itself back and given itself up into His hands, and that is, to give it whatever medicine He pleases, and to administer that which He in His perfect knowledge sees the soul stand in need of for its health and well-being. —*Scupoli.*

To those who know themselves, all things work together for good, and all things seem to be, as they are to them, good. The goods which God gives seem "very good," and God Himself in them, because they know that they deserve them not. The evils which God allows and overrules seem also "very good," because they see in them His loving hand, put forth to heal them of what shuts out God from the soul. They love God intensely, in that He is so good to them in each, and every, the least good, because it is more than they deserve; how much more in the greatest! They love God for every, and each, the very greatest of what seem evils, knowing them to be, from His love, real goods. For He by whom "all the hairs of our head are numbered," and Who "knoweth whereof we are made," directs everything which befalls us in life, in perfect wisdom and love, to the well-being of our souls.—*E. B. Pusey.*

Teach me to live and find my life in thee,
Looking from earth and earthly things away;
Let me not falter, but untiringly
Press on and gain new strength and power each day.

Teach me to live! with kindly words for all,
Wearing no cold, repulsive brow of gloom;
Waiting with patience till Thy call
Summons my spirit to her heavenly home!

—*E. E. Burman.*

### Notes and Queries.

The Editor is always willing to answer in this column, without any fee, all reasonable questions, as soon as possible.

But the following rules must be carefully observed:—

1. Every communication must be accompanied by the name and address of the writer.

2. The question must always bear upon nursing, directly or indirectly.

If an answer is required by letter a fee of half-a-crown must be enclosed with the note containing the inquiry.

*Massage.*

(248) Can you kindly give me some information as to learning the art of massage thoroughly, also Schott's Nauheim treatment? 1. I wish to know if it can be as efficiently learnt in Manchester, as I am nearest to that place, or is it best to go to London or Germany for tuition? Which is cheapest and best in the end of these three places? 2. I would like to know where to go, either in London or abroad, where I might give my services for a time on mutual terms. 3. I presume the reply to my question will be found Saturday week in the "Notes and Queries" of The Hospital "Nursing Mirror."—*H. T. H.*

The Secretary, Society of Trained Masseuses, 12, Buckingham Street, Strand, W.C., will probably be able to recommend a reliable teacher in Manchester. 2. You might hear privately, through friends, of an institution willing to teach you for your services, but do not go anywhere without good references. 3. Queries are published in rotation. It is impossible, however, to fix the date in consequence of the number.

*South Africa.*

(249) Will you kindly inform me how a certified nurse, with excellent medical and surgical testimonials, should proceed, who wishes to go out to the war?—*Greenholme.*

Apply to the Hon. Secretary, the Army Nursing Reserve, 18, Victoria Street, Westminster.

*Lip Reading.*

(250) Would you kindly inform me where I could have lessons in lip reading? I have been a hospital nurse for five years, but was obliged to give it up three years ago on account of deafness.—*M. O. G.*

The Secretary, Training College for Teachers of the Deaf and Dumb, 11, Fitzroy Square, W., would probably be able to advise you in the matter.

*Male Nurses.*

(251) Will you kindly tell me of any institution where they train male nurses?—*E. S. L.*

The only general training in sick nursing for male nurses in England is to be obtained at the National Hospital for the Paralysed and Epileptic, Queen's Square, Bloomsbury, W.C. The majority of male nurses, however, pass through lunatic asylums as attendants, and gain some knowledge of sick nursing in the infirmary wards of these institutions. Course of systematic training and examination are now arranged in many asylums by the Medico-Psychological Association, 11, Chandos Street, Cavendish Square, W.C., of which full particulars may be obtained from the Registrar.

*Nurses' Club.*

(252) Will you kindly tell me of a nurses' club to which I might belong in case of illness?—*M. A. J.*

The best club for nurses, and the only one which provides help in sickness, is the Royal National Pension Fund for Nurses. Apply for full particulars to the secretary, 28, Finsbury Pavement, E.C.

*Young Probationers.*

(253) Will you kindly give me names and address of one or two hospitals that take probationers at the age of 18?—*B. C.*

You should get a copy of the "Nursing Profession: How and Where to Train," price 2s., from the Scientific Press, and apply to the matrons of the institutions which seem most suitable. It is not much use giving the names and addresses of one or two, as there are many points in each case to be considered of which we can know nothing.

*Maternity Training.*

(254) Will you kindly inform me whether, in your opinion, the Hospital for Women and Children, 9, Lupus Street, S.W., is a good place for a woman desiring to be trained as a monthly nurse?—*R. W.*

We have no special information respecting this school, and it is always an advantage to possess a certificate from a well-known hospital.

*Probationer.*

(255) Will you kindly tell me if there is any training home, hospital, or infirmary in London where they take probationers free of charge for one year, and give a certificate?—*L. B.*

St. John's Hospital for Diseases of the Skin, Leicester Square, W.C., appears to be the only one. The certificate is for special work only, and the salary is £15 a year.

*Book on Surgery.*

(256) Can you recommend me a good surgical book, one up to date?—*Anxious.*

"Erichsen's Surgery," or the new work on Surgery by Rose and Carless, are books much used by students.

*Standard Books of Reference.*

"The Nursing Profession: How and Where to Train." 2s. net.
"The Nurses' Dictionary of Medical Terms." 2s. 6d. net.
"Burdett's Series of Nursing Text-Books." 1s. each.
"A Handbook for Nurses." (Illustrated.) 5s.
"Nursing: Its Theory and Practice." New Edition. 3s. 6d.
"Helps in Sickness and to Health." Fifteenth Thousand. 5s.

All these are published by The Scientific Press, Ltd., and may be obtained through any bookseller or direct from the publishers, 28 & 29, Southampton Street, London, W.C.

## Figures and Tables

**Fig. 2. f1:**
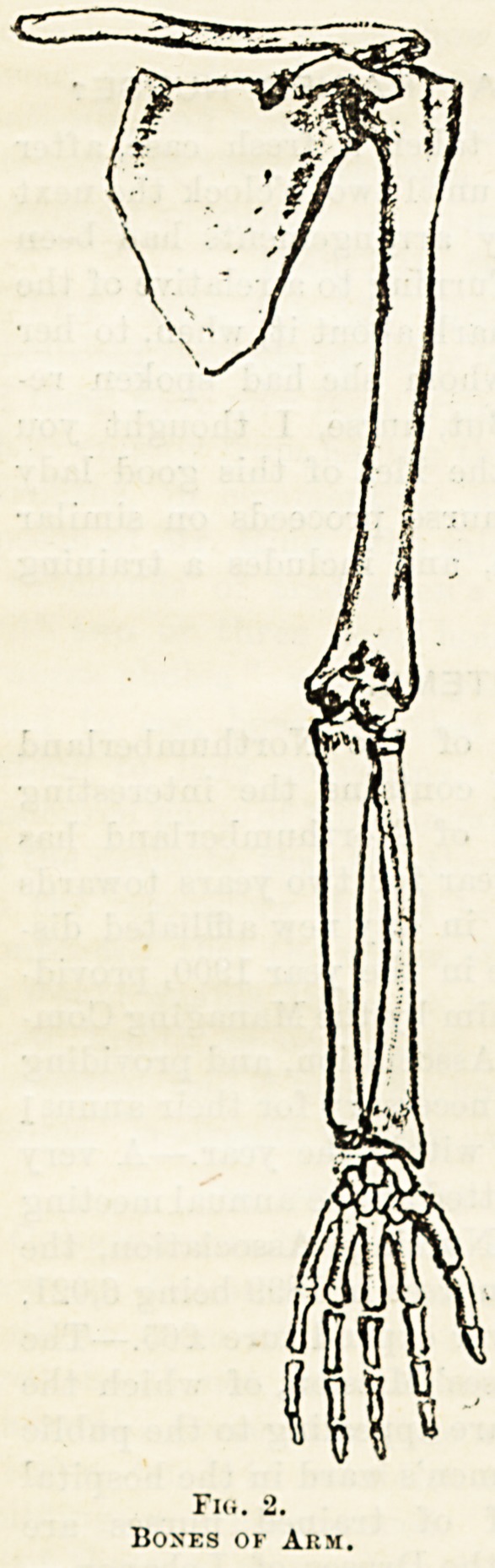


**Fig. 3. f2:**
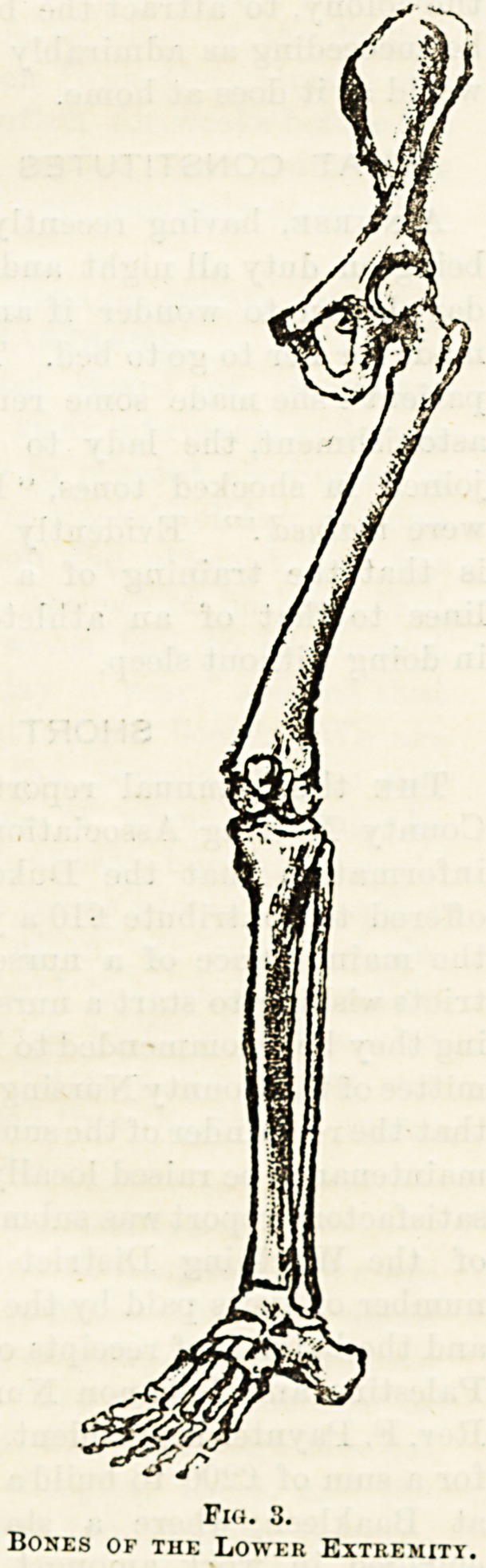


**Fig. 4. f3:**